# Body-limb coordination mechanism underlying speed-dependent gait transitions in sea roaches

**DOI:** 10.1038/s41598-019-39862-3

**Published:** 2019-02-26

**Authors:** Takeshi Kano, Yoshihito Ikeshita, Akira Fukuhara, Akio Ishiguro

**Affiliations:** Research Institute of Electrical Communication, Tohoku University, 2-1-1 Katahira, Aoba-Ward, Sendai, 980-8577 Japan

## Abstract

The sea roach is an isopod with 14 legs; owing to its many degrees of freedom and coordination thereof, it can walk rapidly on rough terrain. Although there likely exists a remarkable decentralized control mechanism that facilitates fast and adaptive locomotion of sea roaches, it still remains elusive. To address this issue, we performed behavioural experiments and revealed that sea roaches often change their gait patterns depending on the locomotion speed. We suggest that the bending of the body trunk in the pitch direction is essential for the gait transitions, and we propose a decentralized control mechanism for body-limb coordination. We demonstrate this with a sea-roach-like robot whose gait transition is achieved by the proposed mechanism. This mechanism has some points in common with control mechanisms proposed for other legged animals. Thus, our findings will help unveil the common principle of legged locomotion and aid the design of multi-legged robots that move like animals.

## Introduction

The sea roach has attracted considerable research attention because first, it exhibits astoundingly agile locomotion while adapting to unstructured environments in real time; it can walk distances of more than 10 times its body length per second (see Results Section). Second, studying sea roaches, which have 14 legs, helps understand how control mechanisms of animals with a small number of legs (e.g., quadrupeds and hexapods) are related to those with a larger number of legs (e.g., myriapods) and helps to elucidate a common principle underlying legged locomotion. Thus, understanding the control mechanism of locomotion in sea roaches will provide a basis for establishing a systematic design method for multi-legged robots that can move fast and adapt their locomotion to various environments.

Autonomous decentralized control is likely a key concept for understanding the fast and adaptive nature of locomotion in sea roaches. In fact, results of several studies indicate that animal locomotion is based on autonomous decentralized control mechanisms, such as biochemical oscillators in true slime molds^1^ or distributed neural networks termed central pattern generators (CPGs) in numerous animals^2,3^. Moreover, the neural network structure of sea roaches^4^ is similar to that of insects and myriapods^5^. However, the core of a decentralized control mechanism of locomotion in sea roaches is still largely unclear.

In this study, we report a novel behavioural finding that provides important insights in the decentralized control mechanism of locomotion in sea roaches. Specifically, we found that sea roaches frequently change their gait patterns depending on the locomotion speed. Speed-dependent gait transition phenomena are well known in other legged animals such as quadrupeds^6^ and hexapods^7^, yet in the case of sea roaches, it has been unclear whether gait patterns depend on locomotion speed^8,9^.

Furthermore, we explored the essential decentralized control mechanism for speed-dependent gait transition in sea roaches using a synthetic approach^10–12^. Assuming that this gait transition is caused by pitch bending of the body trunk, we propose a decentralized control model based on body-limb coordination. We developed a sea-roach-like robot to reproduce speed-dependent gait transitions using the proposed control mechanism. We also discuss the similarity of the proposed control mechanism and control mechanisms in quadruped, hexapod, and myriapod locomotion, which we proposed in previous studies^13–15^, and suggest a common principle underlying legged locomotion.

## Results

### Behavioural Experiments

**Figure 1:**
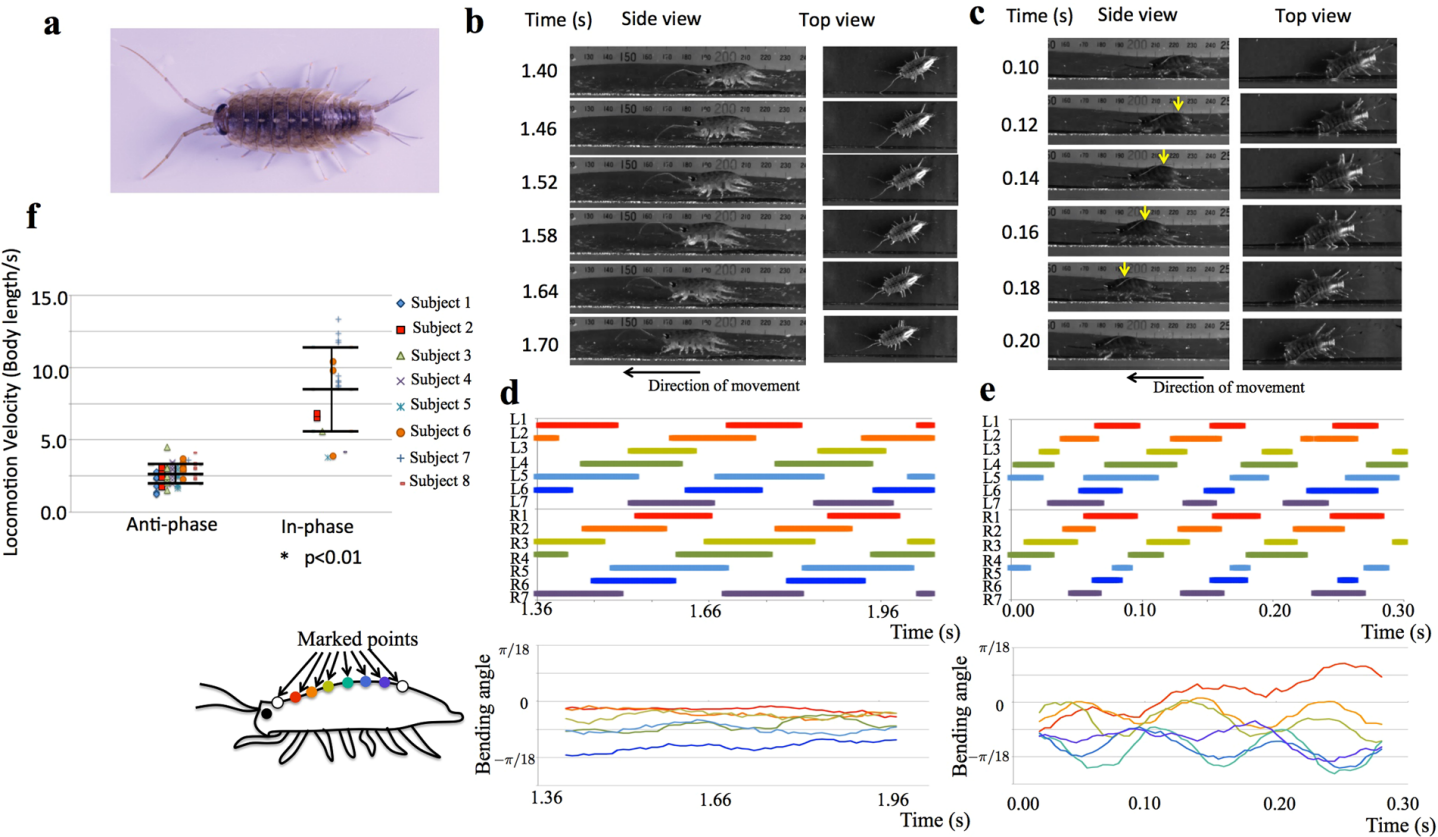
Sea roaches change their gait patterns depending on the locomotion speed. (**a**) Photograph of a sea roach (*Ligia cinerascens*). (**b**) Side and top view of a sea roach during low speed locomotion. (**c**) Side and top view of a sea roach during high speed locomotion. Yellow arrows indicate the parts where the body trunk bends in the pitch direction. (**d**) and (**e**) Gait diagrams corresponding to (**b**) and (**c**), respectively, are shown at the top. Left and right legs are indicated by L and R, respectively, and the legs are numbered from the head. Colour bars indicate the stance phase. The bending angles of the body trunk at several points are shown at the bottom. Bending to the dorsal side was assumed positive. Colours of the curves correspond to the colours shown in the schematic of a sea roach on the left. (**f**) Comparison of the locomotion velocity between the anti-phase and in-phase cases. Bars indicate means and standard deviations (*p < 0.01, Welch test).

We observed the locomotion of eight intact sea roaches (*Ligia cinerascens*) along a lane to assess the locomotion velocity and phase relationships between the legs (see Methods section). Representative photographs of one sea roach’s locomotion in two representative trials are shown in Fig. 1b and c (video clips are provided in the Supplementary Movies 1–4), and the corresponding gait diagrams are shown in Fig. 1d and e, respectively. Moreover, we measured the bending angle of the dorsal surface by analysing video clips (Supplementary Movies 2 and 4), and the results are shown in Fig. 1d and e. We found that the leg density waves propagated from the tail to the head in both cases. The left and right legs are in anti-phase in Fig. 1b and d but are in phase in Fig. 1c and e. Higher velocity was observed when legs were in phase (Fig. 1c and e, 8.54 ×body length/s, on average) than when in anti-phase (Fig. 1b and d, 2.64 ×body length/s, on average). The sea roach bent its body trunk to move effectively when in phase, whereas such body trunk bending was not observed when in anti-phase.

The relationship between gait patterns and the locomotion velocity of all experiments are shown in Fig. 1f (the full dataset is provided in Supplementary Data 1). Locomotion velocity was significantly higher when in phase than when in anti-phase (*p* < 0.01). It is also found that most sea roaches exhibited both the in-phase and anti-phase patterns, whereas a few sea roaches (sea roach 1 and 4) exhibited only the anti-phase pattern. Furthermore, we observed the body trunk bending in the pitch direction several times when left and right legs were in phase.

In summary, sea roaches (*Ligia cinerascens*) frequently change their gait patterns depending on the locomotion speed, which was not found in previous works^8,9^. The bending of the body trunk during fast locomotion suggests that this mechanism likely plays a role in gait transition.

### Model

**Figure 2:**
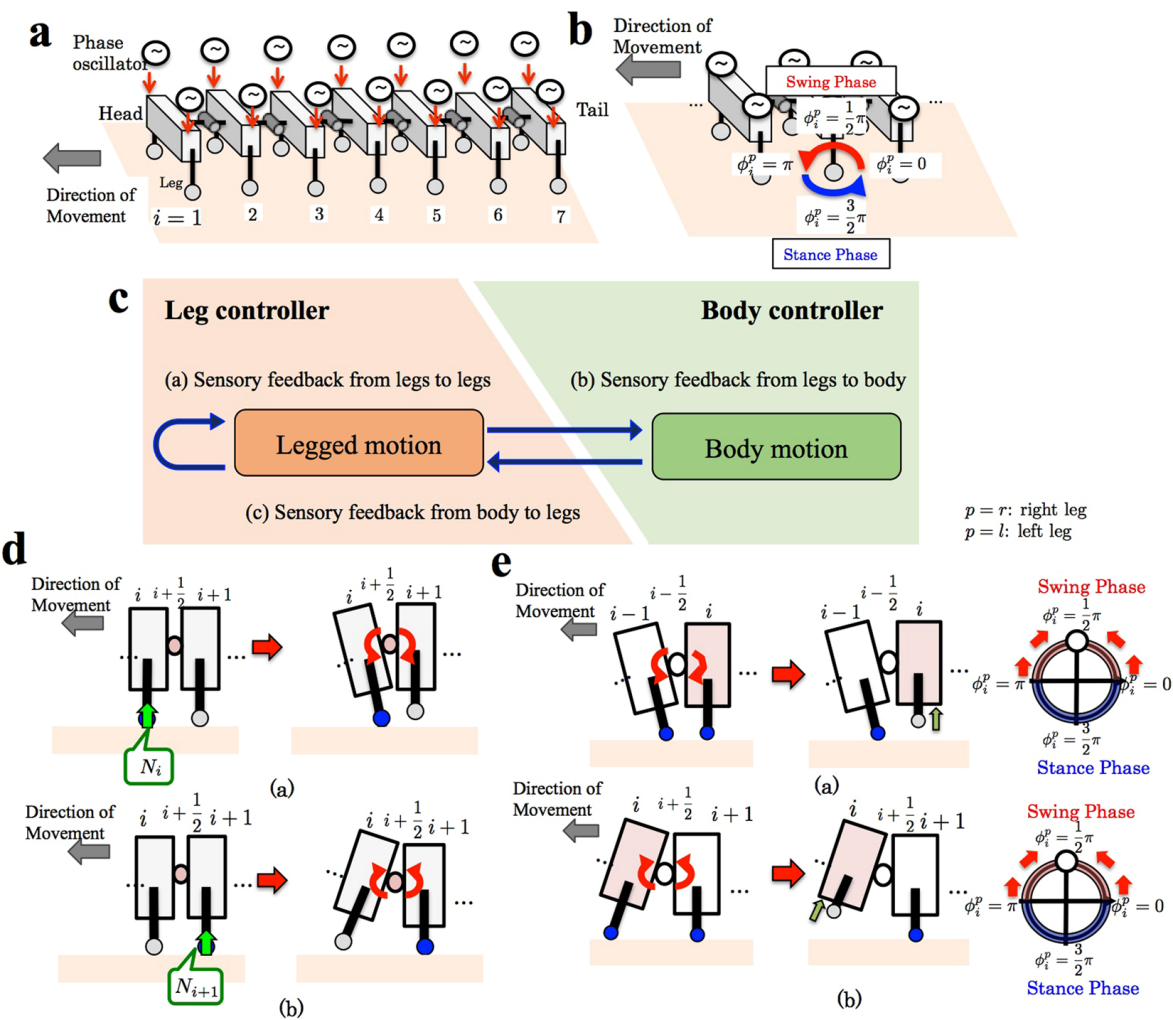
Schematic of the model that implements body-limb coordination mechanism. **(a**) Schematic of the body system. The body consists of seven segments. In each segment, a leg is attached on each side of the body trunk. Pitch joints are implemented between the segments. A phase oscillator is implemented in each leg. (**b)** The relationship between the oscillator phase and the target leg tip position. The leg tends to be in the swing and stance phases when the oscillator phase is between 0 and *π*, and between *π* and 2*π*, respectively. (**c**) Schematic of the control system. Three types of local sensory feedback mechanisms are implemented to achieve body-limb coordination ((a) from legs to legs, (b) from legs to the body, and (c) from the body to legs). (**d**) Schematic of the local sensory feedback from the legs to the body. The pitch joint bends to the dorsal/ventral side when its adjacent posterior/anterior legs receive the ground reaction force. **e**. Schematic of the local sensory feedback from the body to the legs. The phase converges to *π*/2 when its anterior pitch joint bends to the ventral side or when its posterior pitch joint bends to the dorsal side.

The model was based on the results of the behavioural experiments. The body consisted of seven segments (schematic is shown in Fig. 2a). In each segment, legs attached on each side of the body trunk could move in forward–backward and upward–downward directions. A phase oscillator was incorporated in each leg. The target positions of the leg tips of the *i* th leg on the right and left sides were controlled based on the oscillator phases ${\varphi }_{i}^{r}$ and ${\varphi }_{i}^{l}$, respectively. Specifically, the legs tended to be in the swing and stance phases when the oscillator phase was between 0 and *π*, and between *π* and 2*π*, respectively (Fig. 2b). A force sensor attached at the tip of the leg detected the axial component of the ground reaction force. Pitch joints were implemented between the segments. The target angle of the pitch joint connecting the *i* th and (*i* + 1) th segment, ${\bar{\theta }}_{i+1/2}$, was variable.

The behavioural experiments suggested that as locomotion speed increased, the body trunk tended to bend in the pitch direction, which likely affected the phase relationship between left and right legs. Thus, the coordination between the pitch bending of the body and the leg movements is likely a key factor for reproducing the findings of the behavioural experiments. Hence, we hypothesized that sensory information from the legs is fed back into the body trunk as well as into the legs themselves, and sensory information from the body trunk is fed back into the legs (Fig. 2c). Accordingly, the time evolution of the oscillator phases and the target angles of the pitch joints at the body trunk are described as follows:

1 $${\dot{\varphi }}_{i}^{p}=\omega +{f}_{i,a}^{p}+{f}_{i,c}^{p}\,(p=\{\begin{array}{c}r:\mathrm{right}\,{\rm{leg}}\\ l:\mathrm{left}\,{\rm{leg}}\end{array}),$$

2 $${\bar{\theta }}_{i+1/2}={f}_{i+1/2,b}.$$

where *ω* denotes the intrinsic frequency, and ${f}_{i,a}^{p}$, *f*_*i*+1/2,*b*_, and ${f}_{i,c}^{p}$ denote the local sensory feedback terms corresponding to (a), (b), and (c) in Fig. 2c, respectively. Each feedback term is explained in detail below.

According to the previous studies^4,5^, a distributed nervous system of sea roaches^4^ is similar to that in insects^5^, and sea roaches have mechanoreceptors and reflex pathways^16,17^. Thus, we modelled the sensory feedback from a leg to itself and to its nearby legs ${f}_{i,a}^{p}$ in the same manner as that proposed for hexapod locomotion^14^:

3 $${f}_{i,a}^{p}=-\,{\sigma }_{1}{N}_{i}^{p}\,\cos \,{\varphi }_{i}^{p}+{\sigma }_{2}(\frac{1}{{n}_{L}}\sum _{j\in L(p,i)}^{{n}_{L}}{N}_{j}{k}_{j})\cos \,{\varphi }_{i}^{p},$$

where *σ*_1_ and *σ*_2_ are positive constants, ${N}_{i}^{p}$ denotes the ground reaction force acting on the *i* th leg on the side *p*, *L*(*p*, *i*) denotes the class of legs adjacent to the *i* th leg on the side *p* (specifically, (*i* ± 1) th legs on the ipsilateral side and *i* th leg on the contralateral side), *k*_*j*_ = *k*_*f*_, *k*_*c*_, and *k*_*h*_ for the anterior, contralateral, and posterior adjacent legs, respectively, and *n*_*L*_ denotes the number of legs that belong to *L*(*p*, *i*). The oscillator phase is modified when the leg or its nearby legs respond to the ground reaction force. The first term on the right-hand side in Eq. (3) works in such a manner that the phase converges to 3*π*/2 (thus, the leg tends to remain in the stance phase to support the body) in response to the ground reaction force. The second term on the right-hand side in Eq. (3) works in such a manner that the phase converges to *π*/2 (thus, the leg tends to lift off the ground) when its adjacent legs respond to the ground reaction force and support the body.

The sensory feedback from the legs to the body trunk *f*_*i*+1/2,*b*_ is described as

4 $${f}_{i+1/2,b}=\lambda {\omega }^{n}({N}_{i+1}^{l}+{N}_{i+1}^{r}-{N}_{i}^{l}-{N}_{i}^{r}),$$

where *λ* and *n* are positive constants. This term means that the pitch joint bends to the dorsal/ventral side when its adjacent posterior/anterior legs respond to the ground reaction force (Fig. 2d). Thus, we expect that the legs are able to obtain propulsive forces effectively, owing to the bending of the body trunk. The term *ω*^*n*^ in Eq. (4) was implemented because of the observation of the more prominent bending of the body trunk as locomotion speed increased during the behavioural experiments.

The sensory feedback from the body trunk to the leg ${f}_{i,c}^{p}$ is modelled as

5 $${f}_{i,c}^{p}=-\,{\sigma }_{3}\,{\rm{\min }}\,[0,{\tau }_{i-1/2}]\,\cos \,{\varphi }_{i}^{p}+{\sigma }_{4}\,{\rm{\max }}\,[0,{\tau }_{i+1/2}]\,\cos \,{\varphi }_{i}^{p},$$

where *σ*_3_ and *σ*_4_ are positive constants, and *τ*_*i*+1/2_ denotes the torque generated at the (*i* + 1/2) th pitch joint (bending to the dorsal side is taken as positive). Equation (5) works in such a manner that the phase converges to *π*/2 (thus, the leg tends to remain in the swing phase) when its anterior pitch joint bends to the ventral side or when its posterior pitch joint bends to the dorsal side (Fig. 2e). Owing to this feedback mechanism, the legs do not interfere with the bending of the body trunk.

### Robot Experiment

**Figure 3:**
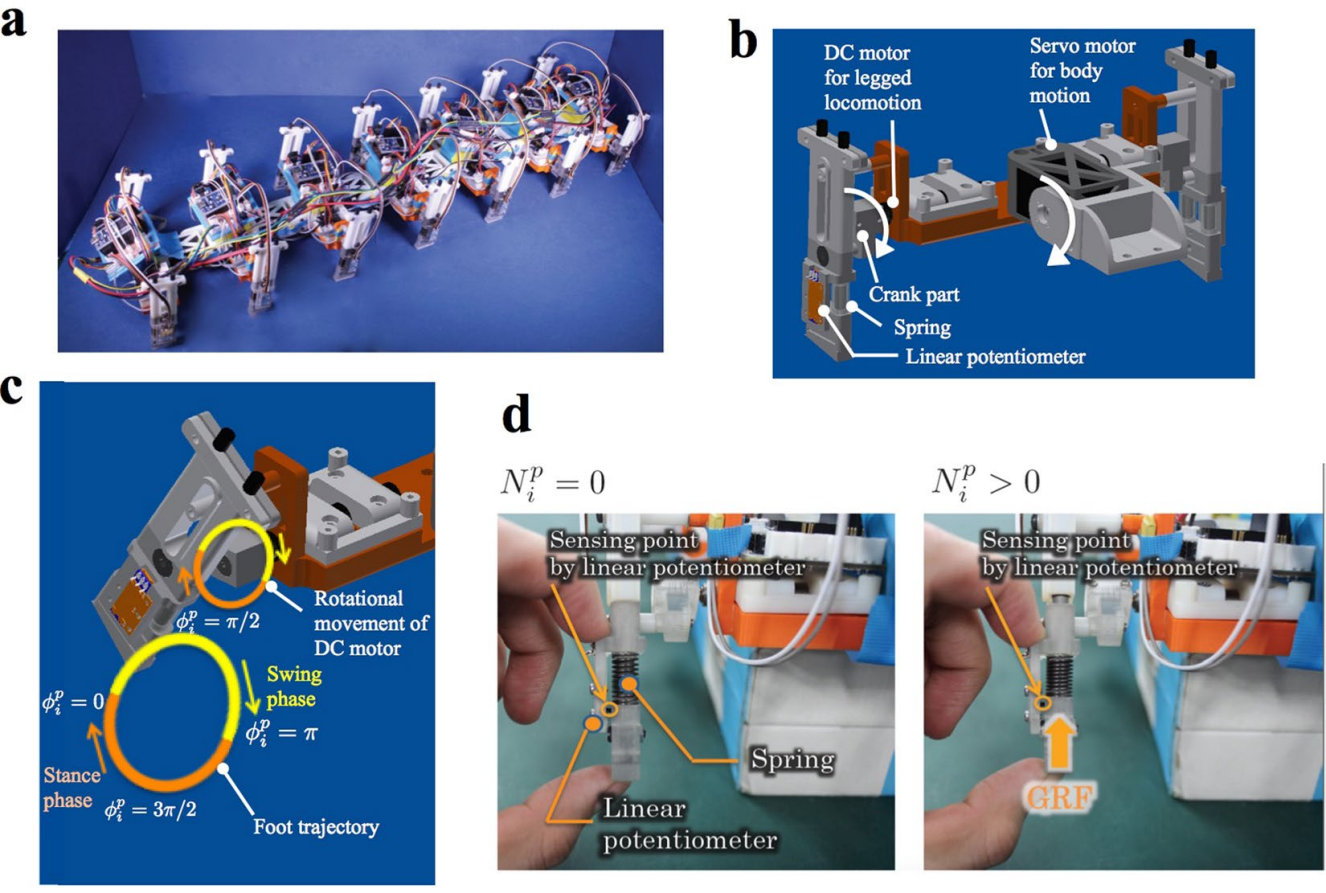
Sea roach-like robot used to validate the proposed model. (**a**) Full view of the robot. (**b**) Detailed structure of a segment. The pitch joint and the legs are operated by a servo motor and DC motors, respectively. (**c**) Trajectory of the leg tip. A crank mechanism was incorporated so that closed trajectory of the leg tip was drawn by driving the DC motor. (**d**) Mechanism for detecting the ground reaction force. The displacement of a spring embedded at the leg tips is detected by a potentiometer.

We developed a sea roach-like robot to test the validity of the proposed model. The robot consisted of seven segments (Fig. 3a), with each segment consisting of the body trunk and two legs (Fig. 3b). Pitch joints were implemented between the segments to bend the body trunk. The legs and the pitch joints were actuated by motors. Each leg moved along the trajectory shown in Fig. 3c. Mechanisms for estimating the torque generated at the pitch joints and the ground reaction forces were implemented (Fig. 3d, details are provided in the Methods section).

We observed the robot’s locomotion with and without feedback from the legs to the body ($$\lambda =1.31\times {10}^{-4}{{\rm{s}}}^{3}/$$$$({{\rm{rad}}}^{2}\cdot {\rm{V}})$$ and $$\lambda =0.00\times {10}^{-4}{{\rm{s}}}^{3}/({{\rm{rad}}}^{2}\cdot {\rm{V}})$$, respectively). The intrinsic angular frequency *ω* was increased from 4.71 rad/s to 9.42 rad/s during each trial. Details of the procedures are provided in the Methods section.

**Figure 4:**
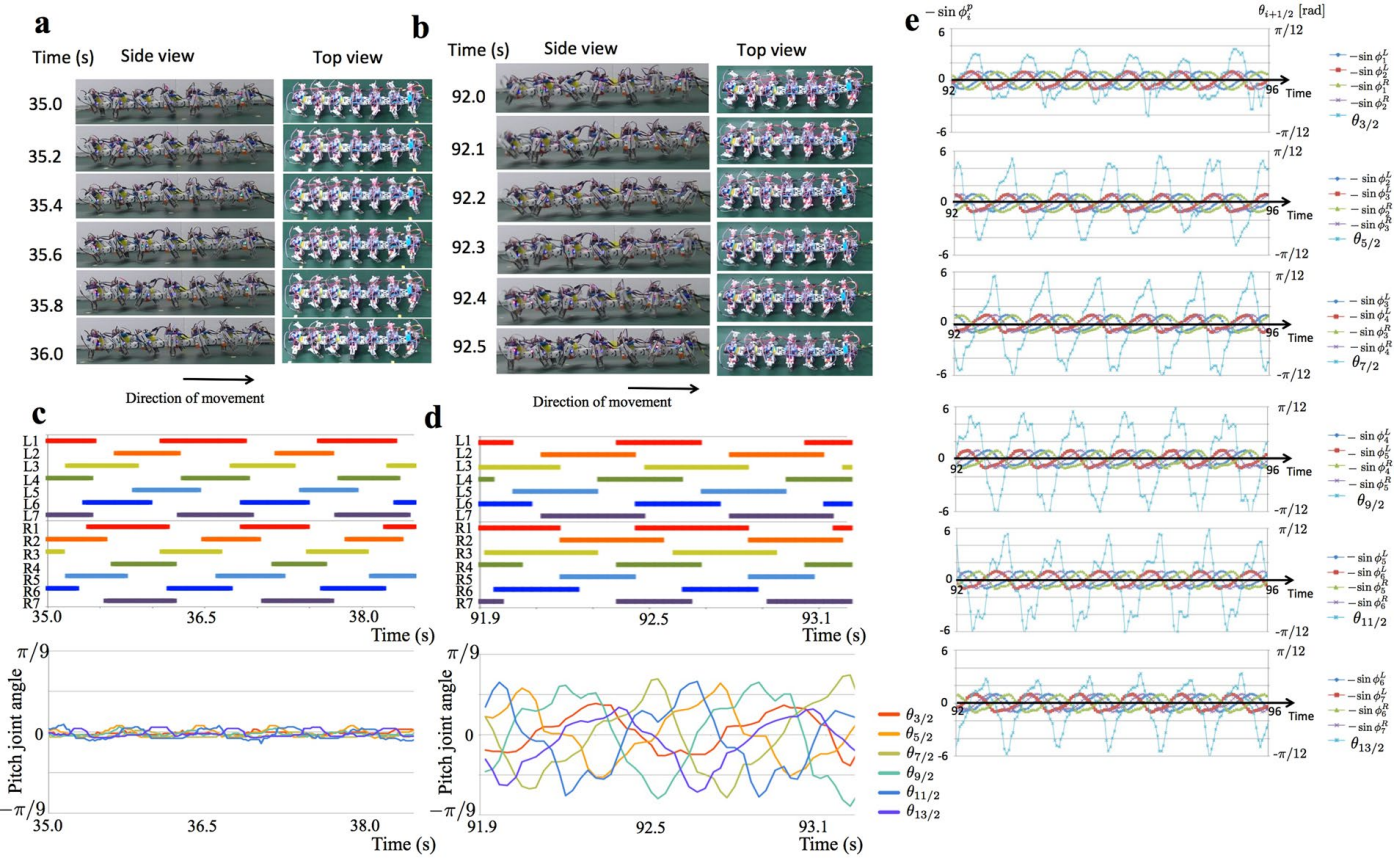
Sea roach gait-velocity relationships emerge in a robot with a body-leg coupling gait control model (**a**) Side and top views of the robot when *ω* = 4.71 rad/s and $\lambda =1.31\times {10}^{-4}{{\rm{s}}}^{3}/({{\rm{rad}}}^{2}\cdot {\rm{V}})$. (**b**) Side and top views of the robot when *ω* = 9.42 rad/s and $\lambda =1.31\times {10}^{-4}{{\rm{s}}}^{3}/({{\rm{rad}}}^{2}\cdot {\rm{V}})$. (**c**) and (**d**) Gait diagrams corresponding to (**a**) and (**b**), respectively, are shown at the top. Left and right legs are denoted by L and R, and the legs are numbered from the head. Colour bars indicate the stance phase. The time evolutions of the pitch joint angles *θ*_*i*+1/2_ are shown at the bottom. (**e**) The time evolutions of the pitch joint angles *θ*_*i*+1/2_ and the oscillator phases when *ω* = 9.42 rad/s and $\lambda =1.31\times {10}^{-4}{{\rm{s}}}^{3}/({{\rm{rad}}}^{2}\cdot {\rm{V}})$.

Figure 4 shows the results for the case with the feedback ($\lambda =1.31\times {10}^{-4}{{\rm{s}}}^{3}/({{\rm{rad}}}^{2}\cdot {\rm{V}})$) (Supplementary Movies 5 and 6). The photographs from the top and side views for a representative trial when *ω* = 4.71 rad/s and *ω* = 9.42 rad/s are shown in Fig. 4a and b, respectively. The corresponding gait diagrams and the time evolution of the pitch joint angles are shown in Fig. 4c and d, respectively. The leg density waves propagated from the tail to the head in both cases. The left and right legs were almost in anti-phase when *ω* = 4.71 rad/s; however, they became close to in-phase when *ω* = 9.42 rad/s. The body trunk bent significantly when *ω* = 9.42 rad/s, while the bending was not clearly observed when *ω* = 4.71 rad/s. These findings generally agree with the behavioural experiments. Figure 4e shows the time evolution of the pitch joint angles and the oscillator phases when *ω* = 9.42 rad/s. The pitch joint angle *θ*_*i*+1/2_ (*i* = 1, 2, 3, 4, 5, 6) was negative when $-\,\sin \,{\varphi }_{i}^{p}$ was positive, and positive when $-\,\sin \,{\varphi }_{i+1}^{p}$ was positive. Thus, the pitch joints bent to the ventral/dorsal side when their anterior/posterior legs contacted the ground.

To evaluate the results quantitatively, we measured the phase relationship between the legs and the locomotion velocity. The phase relationships between adjacent legs on the ipsilateral and contralateral sides were evaluated by using the following indices *R*_*I*_, Φ_*I*_, *R*_*C*_, and Φ_*C*_:

6 $$\begin{array}{c}{R}_{I}{{\rm{e}}}^{{i{\rm{\Phi }}}_{I}}={\langle {\langle {\langle {{\rm{e}}}^{{\rm{i}}({\varphi }_{j-1}^{p}-{\varphi }_{j}^{p})}\rangle }_{{\rm{time}}}\rangle }_{{\rm{legs}}}\rangle }_{{\rm{subjects}}}(p=\{\begin{array}{c}r:{\rm{right}}\,{\rm{leg}}\\ l:{\rm{left}}\,{\rm{leg}}\end{array}),\\ {R}_{C}{{\rm{e}}}^{{i{\rm{\Phi }}}_{C}}={\langle {\langle {\langle {{\rm{e}}}^{{\rm{i}}({\varphi }_{j}^{r}-{\varphi }_{j}^{l})}\rangle }_{{\rm{time}}}\rangle }_{{\rm{legs}}}\rangle }_{{\rm{subjects}}},\end{array}$$

where i denotes an imaginary number and ${\langle \cdots \rangle }_{{\rm{time}}}$, ${\langle \cdots \rangle }_{{\rm{legs}}}$, and ${\langle \cdots \rangle }_{{\rm{subjects}}}$ denote the averages over the time, the legs, and the subjects, respectively (see Methods section). Parameters *R*_*I*_ and *R*_*C*_ characterize the extent of synchronization for adjacent legs on the ipsilateral and contralateral side, respectively. For example, *R*_*I*_ = 1 when ${\varphi }_{j-1}^{p}-{\varphi }_{j}^{p}$ takes a fixed value for all ipsilateral adjacent leg pairs and for all subjects, while it approaches zero as ${\varphi }_{j-1}^{p}-{\varphi }_{j}^{p}$ distributes. Parameters Φ_*I*_ and Φ_*C*_ characterize the average phase difference between adjacent legs on the ipsilateral and contralateral side, respectively.

**Figure 5:**
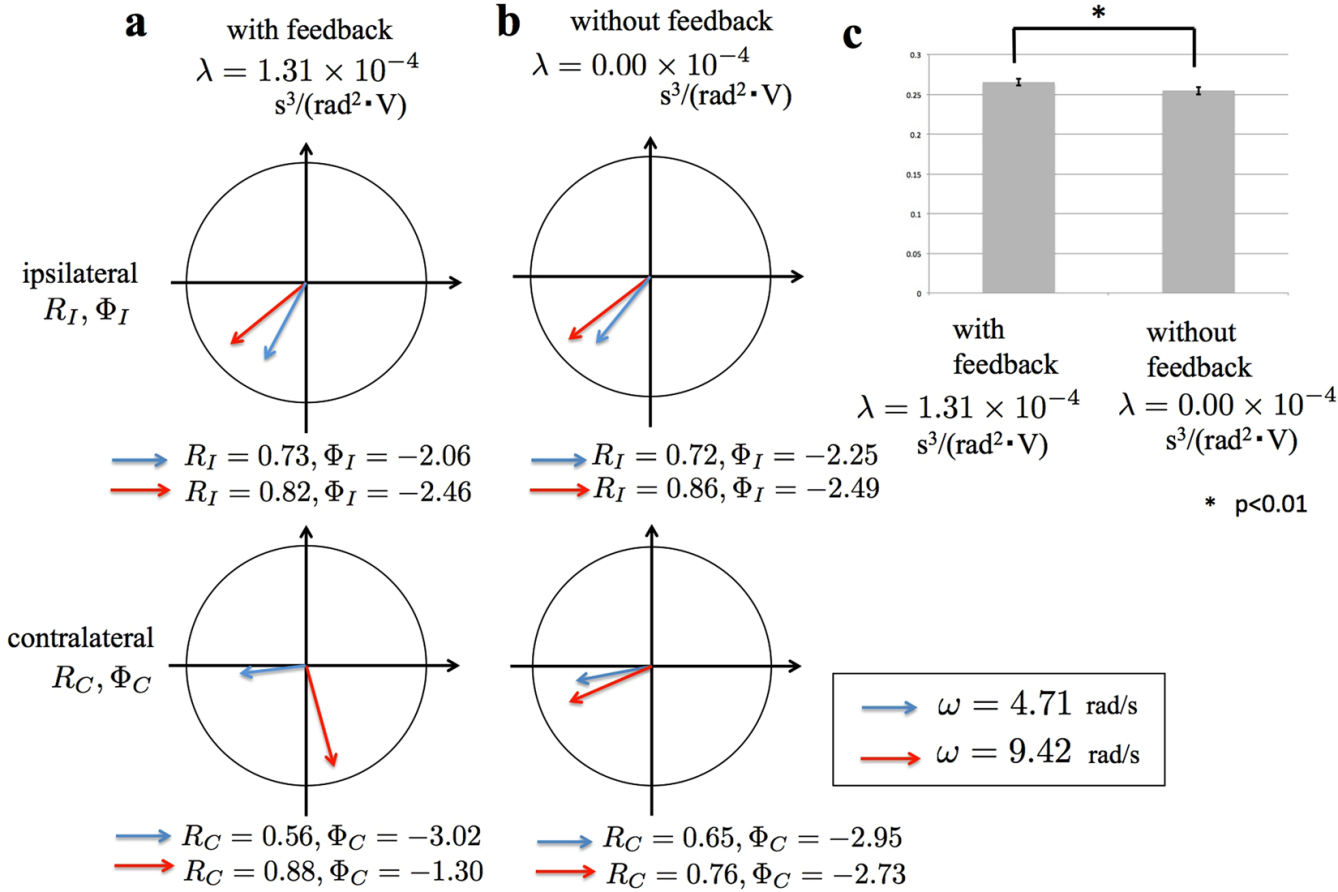
Quantitative data of robot experiments validating the proposed model. The phase relationship between adjacent ipsilateral and contralateral legs in the cases (**a)** with feedback ($\lambda =1.31\times {10}^{-4}{{\rm{s}}}^{3}/({{\rm{rad}}}^{2}\cdot {\rm{V}})$) and (**b**) without feedback ($\lambda =0.00\times {10}^{-4}{{\rm{s}}}^{3}/({{\rm{rad}}}^{2}\cdot {\rm{V}})$). The vectors point (*R*_*I*_*cos*Φ_*I*_, *R*_*I*_*sin*Φ_*I*_)^T^ for the ipsilateral legs and (*R*_*C*_*cos*Φ_*C*_, *R*_*C*_*sin*Φ_*C*_)^T^ for the contralateral legs. Blue and red arrows denote the vectors for *ω* = 4.71 rad/s and *ω* = 9.42 rad/s, respectively. (**c**) Locomotion velocity for *ω* = 9.42 rad/s in the cases with feedback ($\lambda =1.31\times {10}^{-4}{{\rm{s}}}^{3}/({{\rm{rad}}}^{2}\cdot {\rm{V}})$) and without feedback ($\lambda =0.00\times {10}^{-4}{{\rm{s}}}^{3}/({{\rm{rad}}}^{2}\cdot {\rm{V}})$). The bar heights and the error bars indicate means and standard deviations, respectively (*p <0 0.01, *t*-test).

The relevant results are shown in Fig. 5a and b (the dataset is provided in Supplementary Data 2). Parameters *R*_*I*_ and *R*_*C*_ were larger than 0.56 for all cases; thus, the leg movements were well synchronized. When *ω* = 4.71 rad/s, Φ_*I*_ and Φ_*C*_ were around 4*π*/3 and *π*, respectively, and these relations held for the cases both with and without feedback. This result indicated that leg density waves were propagated from the tail to the head with the left and right legs in anti-phase. When *ω* was increased to 9.42 rad/s, Φ_*I*_ did not change significantly, although it slightly shifted toward *π*. In contrast, Φ_*C*_ greatly shifted toward 0 with feedback but remained at around *π* when no feedback was used.

The result for the locomotion velocity when *ω* = 9.42 rad/s is shown in Fig. 5c (the dataset is provided in Supplementary Data 2). Locomotion velocity was significantly higher using a feedback than without feedback (*p* < 0.01).

The above results indicate that the proposed body-limb coordination mechanism plays a crucial role for speed-dependent gait transitions that facilitates effective propulsion.

## Discussion

Our findings provide a plausible explanation for the control mechanism underlying gait transition in sea roaches. At low speeds, the feedback from a leg to itself and its nearby legs ((a) in Fig. 2c) plays a crucial role. This feedback mechanism works so that the leg supports the body. Thus, adjacent legs are not in phase because otherwise they cannot support the body during the swing phase. Hence, the left and right legs are in anti-phase, and the legs form a supporting polygon as in hexapod locomotion^14^. In contrast, at high speeds, the feedbacks from the legs to the body and from the body to the legs ((b) and (c) in Fig. 2c, respectively) are important. Pitch bending of the body trunk is generated owing to the feedback, and it affects the nearby left and right legs equally. For example, when one of the legs on the *i* th segment contacts the ground, the (*i* + 1/2) th pitch joint bends to the ventral side owing to Eq. (4), which modulates the phases of both legs on the (*i* + 1) th segment toward *π*/2 owing to Eq. (5); moreover, the (*i* − 1/2) th pitch joint bends to the dorsal side owing to Eq. (4), which modulates the phases of both legs on the (*i* − 1) th segment toward *π*/2 owing to Eq. (5). Thus, the left and right legs tend to be in phase. Although the proposed body-limb coordination mechanism remains to be elucidated in biological studies and its biological principle is still unknown, we believe that our findings will provide insights for describing animal locomotion in future studies.

A limitation of the present study is that the timing of the body trunk bending of the robot (Fig. 4d and e) was different from that observed in the behavioural experiments where the body trunk tended to bend to the ventral side when the legs were lifted from the ground (Fig. 1e). One possible explanation for this is that the change in the body trunk curvature of real sea roaches is delayed from the timing of actuation owing to weakness of the actuation torque. The difference between sea roaches and the robot regarding stiffness of the body trunk may also affect the timing of body trunk bending. The cause of the discrepancy between the behavioural and robot experiments remains to be investigated.

The contribution of this study is not limited to locomotion in sea roaches. We expect that our findings will be helpful for elucidating the common decentralized control principle underlying legged locomotion. Although neural network structures likely differ in detail between animal species, their basic principles may be similar, and it can be deduced through phenomenological reproduction of animal behaviours using highly abstract models. In fact, the proposed control scheme has several points in common with the control schemes previously proposed for other legged animals^13–15^. For example, the feedback from a leg to itself and its nearby legs ((a) in Fig. 2c) is a part of the control mechanism of hexapod locomotion^14^, whereas feedbacks from the legs to the body and from the body to the legs ((b) and (c) in Fig. 2c, respectively) are part of the control mechanism of quadruped locomotion^13^. Our proposed mechanism is likely also somewhat similar to the control mechanisms of myriapods. In millipede locomotion, leg density waves propagate from the tail to the head with the left and right legs being in phase. Although our previous model^15^ only focused on inter-limb coordination, the body-limb coordination mechanism proposed in this study could also contribute to the emergence of the millipedes’ gait pattern. In contrast, some species of centipedes generate leg density waves that propagate from the head to the tail, with the body trunk undulating in the yaw direction to increase the stride length of the legs^18^. In spite of the difference in the direction of body bending motion between sea roaches and centipedes, *i.e*., pitch and yaw directions, respectively, there may be similar body-limb coordination mechanisms that enable effective propulsion.

Thus, the synthetic approach, *i.e*., the attempt to understand mechanisms using mathematical models and robots, enables us to discuss points that are common or different between control mechanisms of different animal species. Such discussions will benefit researchers in the field of neuroscience and evolution theory because they provide information on how control mechanisms or neural systems may have co-evolved with morphology. Moreover, from an engineering viewpoint, understanding the common principle underlying various legged animals’ locomotion will help establish a systematic design method for legged robots that move like real animals. We propose a plausible body-limb coordination mechanism in which the body bending helps increase the stride length of the legs and thus increase the locomotion speed (Fig. 5c); it can be applied to legged robots with various numbers of legs.

A potential further step would be a reduction in weight and size of the robot to better simulate locomotion in real sea roaches. The extension of the control scheme is another possible improvement; we have observed that real sea roaches increase the amplitude of their leg movement as their locomotion speed increases, and this effect could be incorporated into the current model wherein each leg moves along a fixed trajectory. These improvements will enable the robot to move faster and more efficiently by using the proposed feedback mechanisms.

## Methods

### Behavioural experiments

Eight intact sea roaches (*Ligia cinerascens*) collected at the seashore in Miyagi Prefecture of Japan were used. The body length and weight of the study animals were 30 ± 4 mm and 0.87 ± 0.33 g, respectively. The schematic of the experimental setup is shown in Supplementary Fig. 1. The sea roaches moved along a lane of 68.2 mm width, and ten trials were performed for each sea roach. When the sea roaches did not move voluntarily, we stimulated them by touching the uropods and the dorsal body surface. The locomotion was monitored by two high-speed cameras (DITECT, type HAS-U2) from the side and from above. The resolution of the high-speed camera was 1280 × 768 pixels, and the frame rate was 500 fps.

Because it was difficult to measure the ground reaction forces, the gait diagrams shown in Fig. 1d and e were drawn by manually analysing the snapshots taken by the high-speed cameras. In a few cases a leg was hidden by the body trunk or by other legs and could not be observed from either top or side views. In such cases, the timing of the foot contact or detachment was estimated from its to-and-fro movement. It was also difficult to automate the process of obtaining the phase relationship between the legs. Thus, in Fig. 1f, we evaluated whether the left and right legs were in phase or in anti-phase by carefully looking at the videos taken by the high-speed cameras.

The bending angle of the body trunk shown in Fig. 1d and e was also measured manually because automatic tracking of specific points was difficult. We printed out the snapshots of Supplementary Movies 2 and 4 and marked 8 points equidistantly along the dorsal surface of the body trunk in each snapshot. The points were marked near the proximal ends of the legs (Fig. 1d and e). The marked points were connected by lines, and the angles between adjacent lines were measured by using a protractor. Because the obtained data were variable owing to inaccuracy of hand work, they were approximated by taking moving average over the range of eleven plots.

In the above-mentioned experiments, sea roaches did not tend to move straight in the middle of the lane, and their legs often touched the side walls. Unfortunately, it was inevitable because it seems that they have a habit to approach nearby objects. To examine whether the contact with the side walls affects gait patterns or not, we performed an additional experiment wherein a sea roach (15 mm body length, 0.14 g body weight) moved on a flat surface without side walls, which was monitored from above. Then, we found that it exhibited both anti-phase pattern (3.59 body length per second, Supplementary Movie 7) and in-phase pattern (4.20 body length per second, Supplementary Movie 8). This result indicates that the contact with the side walls is not essential for the emergence of the anti- and in-phase patterns (Fig. 1 and Supplementary Movies 1–4). We cannot exclude a possibility that pitch bending of the body trunk (Fig. 1c and e and Supplementary Movies 1) was caused by the contact with the side wall, because side view images could not be measured owing to technical difficulty in this additional experiment. However, it is suggested (though not fully validated) from our synthetic model that the pitch bending of the body contributes to the gait transition.

### Body structure of the robot

The robot, consisting of seven segments, was 0.71 m long, 0.21 m wide, 0.11 m high, and weighted 2.5 kg (Fig. 3a). Pitch joints were incorporated between the segments. Each segment consisted of the body trunk and two legs (Fig. 3b). A DC motor (Maxon Japan Corporation, DCX10L EB KL 12 V + GPX10 64:1 + ENX10 EASY 32IMP) was incorporated in each leg. The mechanism shown in Fig. 3c converts the rotational motion of the DC motor into an ellipsoidal foot trajectory. Furthermore, a servo motor (Kondo Kagaku, KRS-2572 HV ICS) was incorporated at the centre of each segment to drive the pitch joint (Fig. 3b). The motors were controlled by microcomputers (mbed NXP LPC1768) incorporated in each segment.

Because it was difficult to directly measure the pitch joint torque *τ*_*i*+1/2_, it was calculated from the difference between the target and real angle of the pitch joint, which could be obtained from the servo motor. Hence, in the hardware experiments, the unit of *τ*_*i*+1/2_ in Eq. (5) is rad. Moreover, because it was difficult to directly measure the ground reaction force ${N}_{i}^{p}$, it was expressed by the voltage output of the potentiometer that detects the displacement of the spring implemented at the leg tip (Fig. 3d). Hence, in the hardware experiments, the unit of ${N}_{i}^{p}$ in Eq. (4) is V. To examine whether the ground reaction force is properly estimated by measuring the displacement of the spring, we performed an additional experiment. The experimental setup is shown in Supplementary Fig. 2a. One of the segments was detached from the robot and attached to a slider perpendicular to the ground. Force sensors (Optoforce, OMD-30-SE-100N) were placed on the ground. The left and right legs moved periodically in anti-phase (9.42 rad/s) on the force sensors to measure the displacement of the springs and the ground reaction forces simultaneously (Supplementary Movie 9). The result is shown in Supplementary Fig. 2b. We find that the displacement is large when the ground reaction force is large, although the increase of the displacement often precedes that of the ground reaction force owing to backlash in the force measurement system. Thus, the ground reaction force is roughly estimated by the displacement of the spring.

### Robot experiment

Five trials were conducted under the same experimental conditions. In each trial, the robot was powered on and was then placed on a treadmill. The intrinsic angular frequency *ω* was increased from 4.71 rad/s to 9.42 rad/s after several tens of seconds after the start of the experiment. The other parameter values, which were chosen by trial-and-error, are shown in Supplementary Table 1. To avoid evaluating a transient process before convergence to steady gait patterns, the phase relationship between the legs (Fig. 5a and b) was not evaluated until 10 s after the robot was placed on the ground or the moment when *ω* was increased from 4.71 rad/s to 9.42 rad/s. The locomotion velocity (Fig. 5c) was evaluated for several seconds during which the robot moved straight and steadily.

## Supplementary information

Supplementary Movie 1

Supplementary Movie 2

Supplementary Movie 3

Supplementary Movie 4

Supplementary Movie 5

Supplementary Movie 6

Supplementary Movie 7

Supplementary Movie 8

Supplementary Movie 9

Supplementary Information

Supplementary Data 1

Supplementary Data 2

## References

[1] Takamatsu A (2001). Spatio-temporal symmetry in rings of coupled biological oscillators of *Physarum* plasmodium. Phys. Rev. Lett..

[2] Ijspeert AJ (2008). Central pattern generators for locomotion control in animals and robots: a review. Neural Netw..

[3] Frigon A (2012). Central pattern generators of the mammalian spinal cord. Neuroscientist.

[4] Sakurai A, Yamagishi H (1998). Identification of two cardioacceleratory neurons in the isopod crustacean, Ligia exotica and their effects on cardiac ganglion cells. J. Compar. Biol..

[5] Smarandache-Wellmann CR (2016). Arthropod neurons and nervous system. Curr. Biol..

[6] Owaki D, Ishiguro A (2017). A quadruped robot exhibiting spontaneous gait transitions from walking to trotting to galloping. Sci. Rep..

[7] Beer RD, Quinn RD, Chiel HJ, Ritzmann RE (1997). Biologically inspired approaches to robotics: what can we learn from insects?. Commun. ACM.

[8] Alexander CG (1972). Locomotion in the isopoda crustacean, *Ligia Oceanica* (Linn.). Compara. Biochem. Physiol. A: Physiol..

[9] Walting, L. & Thiel, M. *Functional morphology and diversity: The natural history of the crustacea* (Oxford Univeristy Press, 2013).

[10] Ijspeert AJ (2014). Biorobotics: Using robots to emulate and investigate agile locomotion. Science.

[11] Gravish N, Lauder GV (2018). Robotic-inspired biology. J. Exp. Biol..

[12] Pfeifer, R. & Bongard, J. *How the Body Shapes the Way We Think: A New View of Intelligence* (The MIT Press, Cambridge, MA, 2006).

[13] Fukuhara, A., Koizumi, Y., Suzuki, S., Kano, T., & Ishiguro, A. Minimal model for body-limb coordination in quadruped high-speed running, *Proc. of the 15*^*th*^*Int. Conf. Simulation of Adaptive behavior*, accepted.

[14] Owaki D, Goda M, Miyazawa S, Ishiguro A (2017). A minimal model describing hexapedal interlimb coordination: the Tegotae-based approach. Front. Neurorob..

[15] Kano T, Sakai K, Yasui K, Owaki D, Ishiguro A (2017). Decentralized control mechanism underlying interlimb coordination of millipedes. Bioinsp. Biomim..

[16] Alexander CG (1969). Structure and properties of mechanoreceptors in the pereiopods of *Ligia oceanica* Linn. (crustacea, isopoda). Compara. Biochem. Physiol..

[17] Alexander CG (1970). Studies on the nervous system of an isopod crustacean, *Ligia oceanica*. Compara. Biochem. Physiol..

[18] Manton, S.M. *The Arthropoda: Habits*, *Functional Morphology and Evolution*, (Clarendon Press, 1977).

