# Supplementary material for: Body-limb coordination mechanism underlying speed-dependent gait transitions in sea roaches
Source: Sci Rep. 2019 Feb 26;9:2848. doi: 10.1038/s41598-019-39862-3 (PMC6391416; doi:10.1038/s41598-019-39862-3)
Supplement: Supplementary file 10 — Supplementary Information [file 41598_2019_39862_MOESM10_ESM.docx]

**Body-limb coordination mechanism underlying speed-dependent gait transitions in sea roaches**

Takeshi Kano^1*^, Yoshihito Ikeshita^1^, Akira Fukuhara^1^, and Akio Ishiguro^1^

^1^Research Institute of Electrical Communication, Tohoku University, 2-1-1 Katahira, Aoba-Ward, Sendai 980-8577, Japan.

**【Supplementary　Figure】**

| **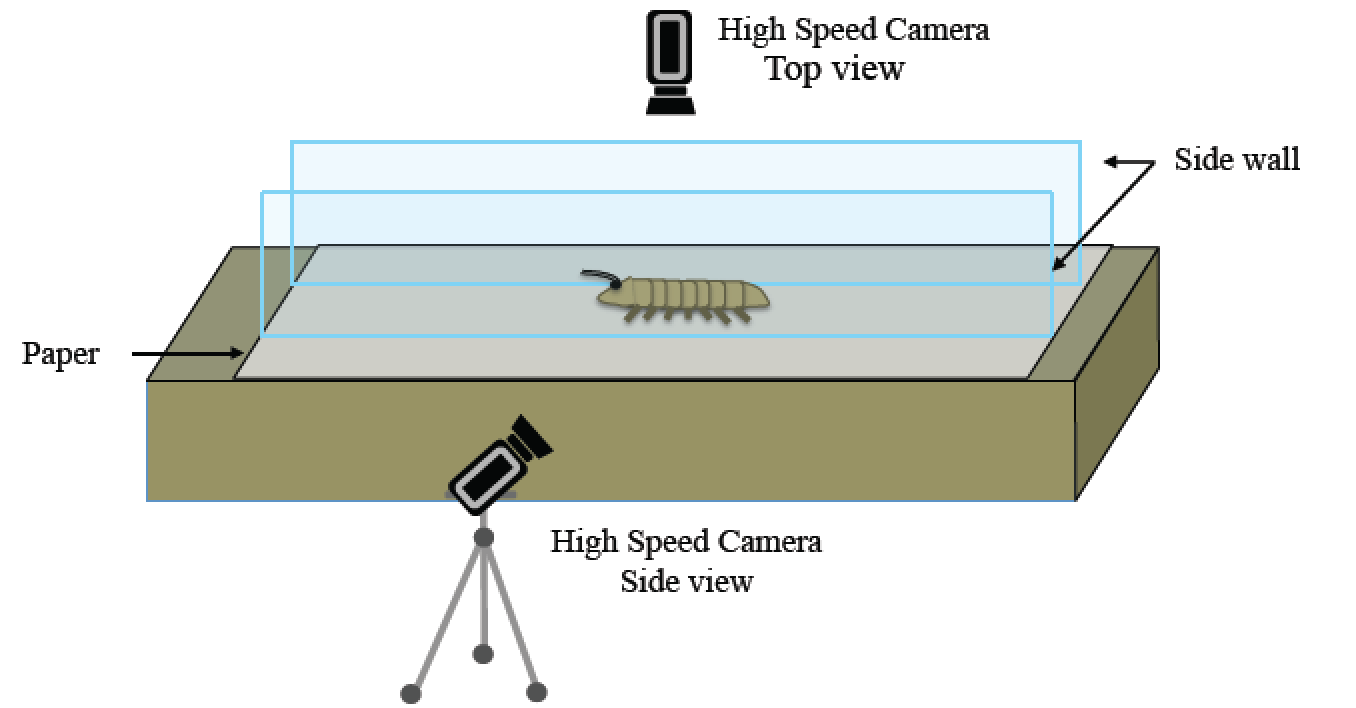** |
| --- |
| **Supplementary Figure 1 \| Experimental setup**  Sea roaches were constrained to move along a lane. The locomotion was monitored by two high speed cameras: one is from above and one from the side. |

| **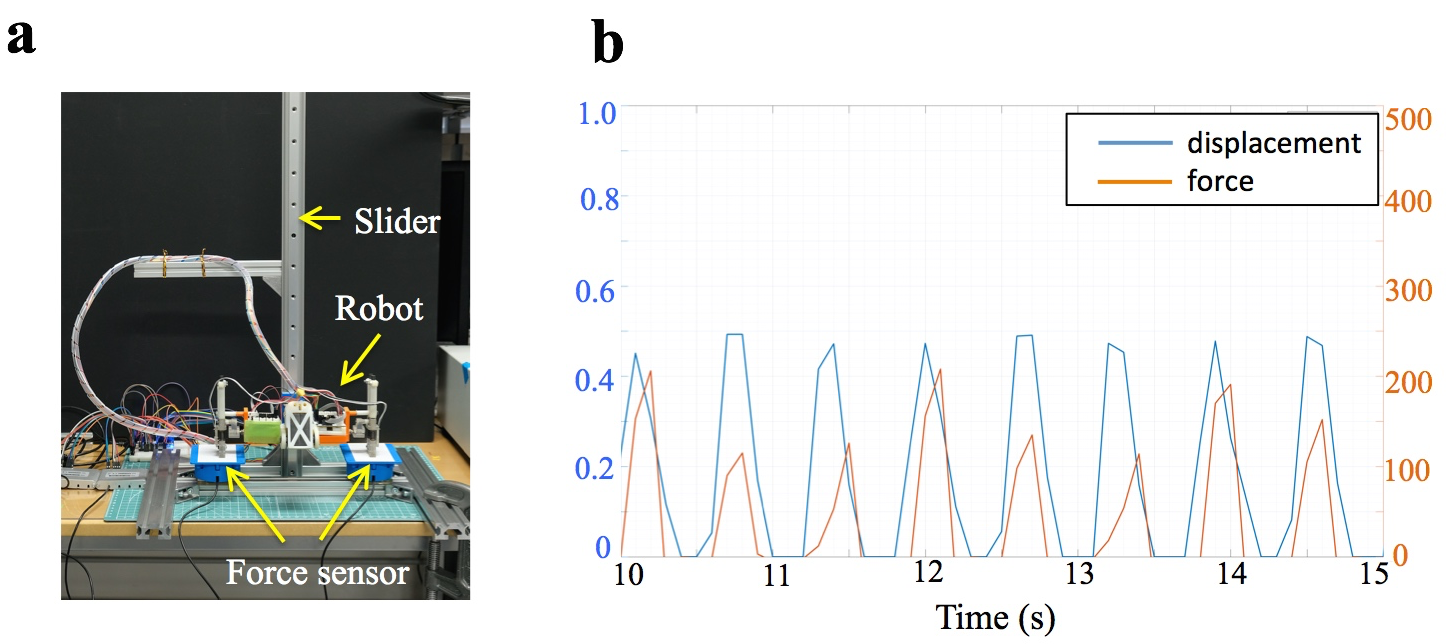** |
| --- |
| **Supplementary Figure 2 \| Comparison between the ground reaction force and the displacement of the spring**  **a.** Experimental Setup. **b.** Experimental result. The orange and blue lines denote the sensor values of the force and the displacement of a spring of the right leg, respectively. |

**【Supplementary Table】**

| **Supplementary Table 1 \| Parameter values used in the robot experiments**   \| Parameter \| Value \| Unit \| \| --- \| --- \| --- \| \| **$\sigma_{1}$** \| 2.5 \| rad/(s$\cdot$V) \| \| **$\sigma_{2}$** \| 30.0 \| rad/(s$\cdot$V) \| \| **$\sigma_{3}$** \| 47.5 \| /s \| \| **$\sigma_{4}$** \| 47.5 \| /s \| \| **$n$** \| 3 \| - \| \| **$k_{f}$** \| 0.85 \| - \| \| **$k_{c}$** \| 0.05 \| - \| \| **$k_{h}$** \| 0.10 \| - \| |
| --- | --- | --- | --- | --- | --- | --- | --- | --- | --- | --- | --- | --- | --- | --- | --- | --- | --- | --- | --- | --- | --- | --- | --- | --- | --- | --- | --- |

**【Supplementary Data】**

**Supplementary Data 1 | Dataset for behavioural experiments**

**Supplementary Data 2 | Dataset for robot experiments**

**【Supplementary Movie】**

**Supplementary Movie 1 | Slow locomotion of a sea roach (Figs. 1b and d; top view)**

**Supplementary Movie 2 | Slow locomotion of a sea roach (Figs. 1b and d; side view)**

**Supplementary Movie 3 | Fast locomotion of a sea roach (Figs. 1c and e; top view)**

**Supplementary Movie 4 | Fast locomotion of a sea roach (Figs. 1c and e; side view)**

**Supplementary Movie 5 | Robot experiment when** $\lambda=1.31\times10^{-4}\text{ s}^{3}/(\text{rad}^{2}\cdot\text{V})$ **(Fig. 4; top view)**

**Supplementary Movie 6 | Robot experiment when** $\lambda=1.31\times10^{-4}\text{ s}^{3}/(\text{rad}^{2}\cdot\text{V})$ **(Fig. 4; side view)**

**Supplementary Movie 7 | Slow locomotion of a sea roach on a flat surface without side walls**

**Supplementary Movie 8 | Fast locomotion of a sea roach on a flat surface without side walls**

**Supplementary Movie 9 | Experiment for the comparison between the ground reaction force and the displacement of the spring**
